# Ground Control Point-Free Unmanned Aerial Vehicle-Based Photogrammetry for Volume Estimation of Stockpiles Carried on Barges

**DOI:** 10.3390/s19163534

**Published:** 2019-08-13

**Authors:** Haiqing He, Ting Chen, Huaien Zeng, Shengxiang Huang

**Affiliations:** 1 School of Geomatics, East China University of Technology, Nanchang 330013, China; 2 School of Water Resources & Environmental Engineering, East China University of Technology, Nanchang 330013, China; 3 National Field Observation and Research Station of Landslides in the Three Gorges Reservoir Area of Yangtze River, China Three Gorges University, Yichang 443002, China; 4 School of Geodesy and Geomatics, Wuhan University, Wuhan 430079, China

**Keywords:** volume estimation, unmanned aerial vehicle (UAV), photogrammetry, structure-from-motion (SfM), semi-global matching (SGM)

## Abstract

In this study, an approach using ground control point-free unmanned aerial vehicle (UAV)-based photogrammetry is proposed to estimate the volume of stockpiles carried on barges in a dynamic environment. Compared with similar studies regarding UAVs, an indirect absolute orientation based on the geometry of the vessel is used to establish a custom-built framework that can provide a unified reference instead of prerequisite ground control points (GCPs). To ensure sufficient overlap and reduce manual intervention, the stereo images are extracted from a UAV video for aerial triangulation. The region of interest is defined to exclude the area of water in all UAV images using a simple linear iterative clustering algorithm, which segments the UAV images into superpixels and helps to improve the accuracy of image matching. Structure-from-motion is used to recover three-dimensional geometry from the overlapping images without assistance of exterior parameters obtained from the airborne global positioning system and inertial measurement unit. Then, the semi-global matching algorithm is used to generate stockpile-covered and stockpile-free surface models. These models are oriented into a custom-built framework established by the known distance, such as the length and width of the vessel, and they do not require GCPs for coordinate transformation. Lastly, the volume of a stockpile is estimated by multiplying the height difference between the stockpile-covered and stockpile-free surface models by the size of the grid that is defined using the resolution of these models. Results show that a relatively small deviation of approximately ±2% between the volume estimated by UAV photogrammetry and the volume calculated by traditional manual measurement was obtained. Therefore, the proposed approach can be considered the better solution for the volume measurement of stockpiles carried on barges in a dynamic environment because UAV-based photogrammetry not only attains superior density and spatial object accuracy but also remarkably reduces data collection time.

## 1. Introduction

Measurement of stockpile volumes, which comprise various materials, such as coal, gypsum, chip, gravel, dirt, rock, quarry, and mine tailings, is an essential task in the construction and mining industry [1,2,3,4]. Conventional methods of volume measurement include the trapezoidal and cross-sectioning methods [5]. These methods generally assume that the geometric shape is regular (e.g., rectangular, triangular prisms, and trapezoidal) and can be modeled using ideal mathematical models, such as trapezoidal, Simpson-based, cubic spline, and cubic Hermite formulas. Moreover, these methods require the collection of three-dimensional (3D) points that appropriate distribution and density for volume calculation [6]. However, in most instances, the surface of stockpiles is not a regular geometric shape; therefore, ideal mathematical models cannot accurately represent the real shapes of stockpiles [2].

Various methods are available to estimate the volume of stockpiles with irregular geometry. On the basis of the density of 3D measured points, these methods are divided into two categories, i.e., (1) sparse point and (2) dense point cloud-based methods. Global positioning system (GPS) and total station instruments are often used to collect certain 3D sparse points for the modeling of a stockpile surface and to calculate the volume by interpolating points [7,8]. Although point-sampled methods can handle the complex surface of stockpiles, the volume cannot be estimated properly if the number of 3D points is small [2]. The surface of the stockpiles is also presented depending on the distribution of 3D points and interpolation methods [5,9]. Moreover, ports can unload stockpiles carried on barges without time delay. Meanwhile, the aforementioned methods are usually labor intensive and time consuming and therefore unsuitable for a rapid volume measurement of stockpiles carried on barges. Close-range photogrammetry is commonly used to estimate the volume of stockpiles by accurately measuring 3D points from overlapping images taken from different perspectives using a close-range camera. Using such a method is fast, productive, and economical. In many cases, 3D points on the surface of stockpiles can still be obtained even when the stockpile is inaccessible or subject to safety restrictions. Yakar et al. [10] investigated the performance of close-range photogrammetry for volume computation in an excavation site and verified its applicability to volume calculations. Fawzy et al. [3] used digital close-range photogrammetry as an alternative to traditional methods using total station instruments for volume calculation, which yielded a better result (97.21%) compared with that of the traditional method. Abbaszadeh et al. [11] compared close-range photogrammetry using a nonprofessional camera with traditional field surveying for volume estimation; they reported that an accurate volume estimation can be achieved using close-range photogrammetry due to its ability to measure negative slopes and berms. In addition, close-range photogrammetry can measure 3D points using a non-contacting manner that reduces the risks associated with exposing surveyors to danger during on-site operations. However, finding suitable camera placements to capture overlapping images from different perspectives is difficult around stockpiles carried on barges with a narrow space.

Unmanned aerial vehicle (UAV)-based photogrammetry, which is flexible and low-cost, can work in a close-range domain, can generate high-resolution and dense 3D point clouds, and can be used for the aerial mapping of 3D terrain models [12,13,14]; thus, it has been widely accepted for the volume estimation of stockpiles on land [15,16,17,18]. UAV-based photogrammetry can acquire typically high-resolution aerial stereo images at low-altitude positions and reconstruct the 3D surface of a stockpile [17]. Previous studies have confirmed the accuracy of 3D modeling using UAV-based photogrammetry [19,20]. High-resolution remote sensing images with a fine ground sampling distance offer an opportunity to describe irregular stockpiles in detail, and they can be used to create precise 3D surface models (i.e., stockpile-covered and stockpile-free surface models) using structure-from-motion (SfM) and dense matching. Several ground control points (GCPs) are typically measured using a real-time kinematic global positioning system to georeference the UAV-based photogrammetric point clouds. However, compared with the abovementioned methods, which rely on discretely distributed measuring points obtained from GPS or total station instruments for stockpile surface modeling, UAV-based photogrammetry can provide a more accurate solution to estimate the volume of stockpiles. In addition, UAVs allow surveyors to collect overlapping images far away from stockpiles instead of climbing them. In this manner, surveyors are not exposed to danger during on-site operations. In addition, terrestrial laser scanning (TLS) has become a popular tool for obtaining the 3D points of a terrain surface [21,22]. TLS-based methods are also widely used to measure the volume of stockpiles because they can rapidly capture dense 3D point clouds for the modeling of irregularly shaped stockpile surfaces [23,24]. However, TLS-based methods still need surveyors to walk around the boundaries of stockpiles at the edge of the vessel or climb up stockpiles to afford full coverage of the surface. LiDAR sensors with small size and light weight, such as a high-definition (HDL)-32E LiDAR sensor, can be mounted on UAV for airborne laser scanning (ALS), which is available to collect 3D point clouds when the barges are basically stationary and motionless; otherwise, ALS-based methods cannot be used to reconstruct the surface of stockpiles when barges are moving or shaking. Meanwhile, a laser instrument is far more expensive than low-cost drones, e.g., DJI UAVs. Therefore, UAV-based photogrammetry is more applicable to the volume estimation of stockpiles compared with TLS-based methods.

At present, measuring the volume of stockpiles under the circumstance of a moving or shaking barge, i.e., a dynamic environment, is needed. In other words, compared with similar studies for the absolute orientation in the applications of UAV-based photogrammetry [19,20], UAV imaging and GCP measurement may be needed given the relative motion between the barge and the background. A stable framework for collecting the GCPs to georeference the generated digital surface model of stockpiles carried in a dynamic environment may not be provided. Therefore, studies on the use of UAV-based photogrammetry for the volume measurement of stockpiles under barge movement have been rarely reported. Although photogrammetry software, such as Pix4D and Agisoft PhotoScan, can also estimate volume without GCPs [25,26], the cases applied by the software could be only applicable to the volume estimation on land and are quite different with the case of volume estimation of stockpiles carried on barges. Specifically, the stockpile-free surface is typically not a plane but a complex irregular surface, thus measuring volume above a plane using photogrammetry software such as Agisoft PhotoScan is unavailable to estimate the volume of stockpiles carried on barges, and still requires a unified reference to align stockpile-covered and stockpile-free surface models for volume estimation. On this basis, an accurate and efficient approach using GCP-free UAV photogrammetry is proposed in this study to estimate the volume of a stockpile carried on a barge under a dynamic environment. An indirect absolute orientation based on the geometry of the vessel is used to establish a custom-built framework that can provide a unified reference between stockpile-covered and stockpile-free surface models. In addition, UAV images cover a large proportion of water, which is typically characterized as weak texture and variable undulation. As a result, the water around a barge becomes meaningless for the surface model of the barge. Thus, a region of interest (ROI) within UAV images is extracted to improve the performance of image matching. Particularly, a coarse-to-fine matching strategy is initially used to determine the corresponding points among overlapping images via the scale-invariant feature transform (SIFT) algorithm [27] and the subpixel Harris operator [28]. Then, SfM and semi-global matching (SGM) algorithms [29] are used to recover the 3D geometry and generate the dense point clouds of stockpile-covered and stockpile-free surface models. In turn, these dense point clouds are transformed into a custom-built framework using a rotation matrix that consists of tilt and plane rotations. Lastly, the volume of the stockpile is estimated by multiplying the height difference between the stockpile-covered and stockpile-free surface models by the size of the grid that is defined using the resolution of these models.

The main contribution of this study is to propose an approach using GCP-free UAV-based photogrammetry that is particularly suitable to estimate the volume of stockpiles carried on barges in a dynamic environment. In this approach, the adaptive aerial stereo image extraction, which helps to capture sufficient overlaps for the photogrammetric process from UAV video, and simple linear iterative clustering (SLIC) algorithm, is used to generate a ROI for improving the performance of image matching by excluding water intervention. In particular, a custom-built framework instead of prerequisite GCPs is defined to provide the alignment between stockpile-covered and stockpile-free surface models.

The remainder of this paper is organized as follows: In Section 2, the two study areas and the materials are introduced. In Section 3, the proposed approach using UAV-based photogrammetry is described in detail. In Section 4, comparative experimental results are presented in combination with detailed analysis and discussion. In Section 5, the conclusion of this study and possible future works are discussed.

## 2. Study Area and Materials

### 2.1. Test Site

**Figure 1:**
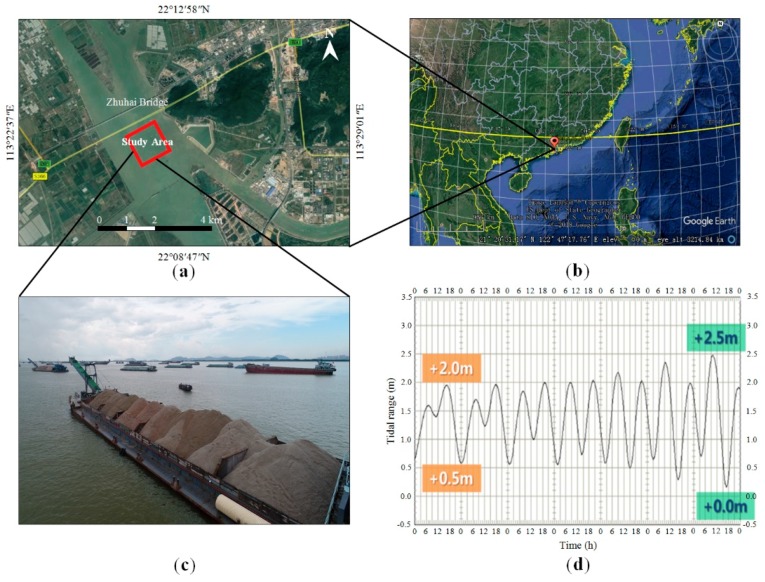
Test site downstream of the Zhuhai Bridge, Southern China: (**a**) the study area that includes several barges; (**b**) the geospatial location described by Google Earth; (**c**) on-site several barges; (**d**) a tidal change plot in the test site.

The study area is located downstream of the Zhuhai Bridge (22°09′28″N, 113°25′56″E) and covers approximately 1.5 km^2^; it is approximately 14 km southeast of Macau, China (Figure 1a,b). The stockpiles consist of sand and gravel (Figure 1c), which are used for the construction of the third runway of the Hong Kong International Airport with a reclamation area over 600 ha. This project includes land formation, construction of sea embankments outside the land, foundation reinforcement, installation of monitoring and testing equipment, and construction of a drainage system.

**Figure 2:**
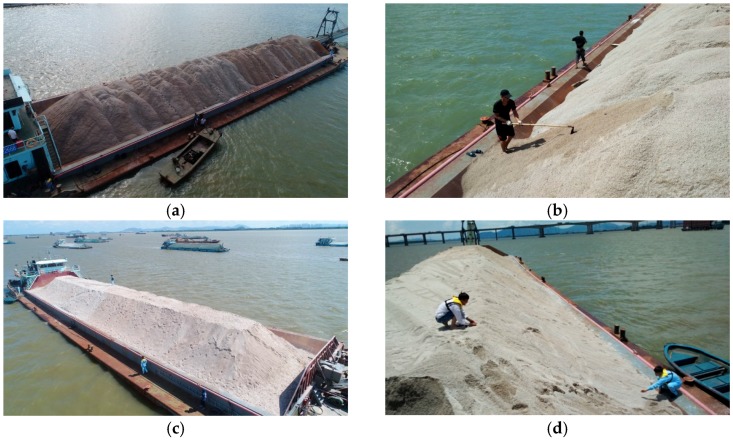
Volume measurement using the traditional method: (**a**) stockpile carried on a barge; (**b**) manual operation for reshaping the surface of the stockpile; (**c**) stockpile with a trapezoidal surface through the reshaping of (**b**); (**d**) volume measurement using a tool, e.g., measuring tape.

The construction area of this project is characterized by a slow rising tide and quick ebb tide, which are caused by the influence of the surrounding topography. Furthermore, the flat tide lasts a long time, and the tidal range is between 0.2 and 2.5 m, as shown in Figure 1d. The water flow velocity in the middle part of the construction area is relatively slow, and the velocity gradually decreases from north to south. Moreover, the water depth of the construction area is shallow in the south and deep in the north. The shallow mud surface is the water area near the south and north sides of the airport land area with an elevation of approximately −2.0 m, whereas the deep mud surface is the northeast water area with an elevation between −5.4 and −7.1 m. The volume of the reclamation area, which is mainly filled with sand, is approximately 90 million m^3^. Traditionally, as shown in Figure 2, the stockpile needs to be reshaped into a regular shape, e.g., trapezoid. The volume of stockpiles carried on barges is usually identified by field measurements using measuring tools, e.g., ruler or ranging instrument. However, some problems, such as low accuracy, low efficiency, numerous surveyors needed, large human error, difficulty in monitoring, and easy divergence with the sand supplier, arise when the traditional method is used. In this case, as shown in Figure 1c, placing the instruments (e.g., GPS, prism, and total station) within the narrow space of barges or on the surface of stockpiles to measure volume is difficult. To find an alternative to the traditional manual volume measurement, UAV photogrammetry and laser scanning are compared and evaluated in terms of indicators, such as accuracy, efficiency, cost, and working conditions.

### 2.2. Field Measurements

**Figure 3:**
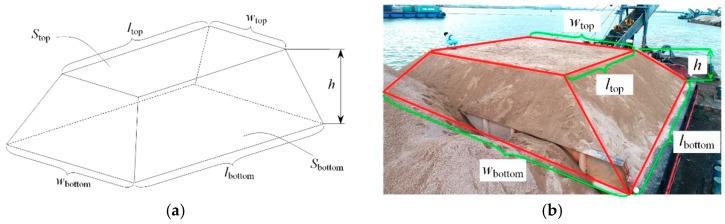
Stockpile with a regular trapezoid: (**a**) model of a stockpile with a regular trapezoid above the flat surface of the vessel; (**b**) a small stockpile with a regular trapezoid on a barge.

The volume of stockpiles carried on barges should be measured at the test site before unloading the stockpiles into the construction area. In this study, the traditional measuring method and laser scanning were compared with the proposed method in June 2018. These experiments were performed under good weather conditions, e.g., sunny and windless. The field measurements include three parts:

(1) For the traditional method, as shown in Figure 2, a labor-intensive operation is performed to reshape the surface of a stockpile into a regular trapezoid. It requires four people to perform the task in approximately 2 h. The measuring tape is used to measure the widths and lengths of the top and the bottom. Thus, the volume $V_{\mathrm{stockpile}}$ of stockpiles with a regular trapezoid shown in Figure 3a can be calculated using the following formula:

(1) $$\begin{array}{l} V_{\mathrm{stockpile}}=V_{\mathrm{stockpile}}^{\mathrm{down}}+V_{\mathrm{stockpile}}^{\mathrm{up}},\\ V_{\mathrm{stockpile}}^{\mathrm{up}}=\left[S_{\mathrm{top}}+S_{\mathrm{bottom}}+\left(w_{\mathrm{top}}+w_{\mathrm{bottom}}\right)\left(l_{\mathrm{top}}+l_{\mathrm{bottom}}\right)\right]\frac{h}{6},\\ S_{\mathrm{top}}=w_{\mathrm{top}}l_{\mathrm{top}},\\ S_{\mathrm{bottom}}=w_{\mathrm{bottom}}l_{\mathrm{bottom}}, \end{array}$$

where $V_{\mathrm{stockpile}}^{\mathrm{down}}$ and $V_{\mathrm{stockpile}}^{\mathrm{up}}$ are the volumes of stockpile under and above the flat surface of the vessel, respectively; $V_{\mathrm{stockpile}}^{\mathrm{down}}$ is a constant; $S_{\mathrm{top}}$ and $S_{\mathrm{bottom}}$ are the areas of the top and bottom planes, respectively; $w_{\mathrm{top}}$ and $l_{\mathrm{top}}$ are the width and length of the top plane, respectively; $w_{\mathrm{bottom}}$ and $l_{\mathrm{bottom}}$ are the width and length of the bottom plane, respectively. However, the trapezoid reshaped through manual operation is seldom a perfectly regular shape, and the error between the calculated result and the real volume of the stockpile cannot be ignored. To calculate the exact volume of the stockpile as accurately as possible, the stockpile is partitioned into several small trapezoids that can be considered for reshaping. A small trapezoid is shown in Figure 3b.

**Figure 4:**
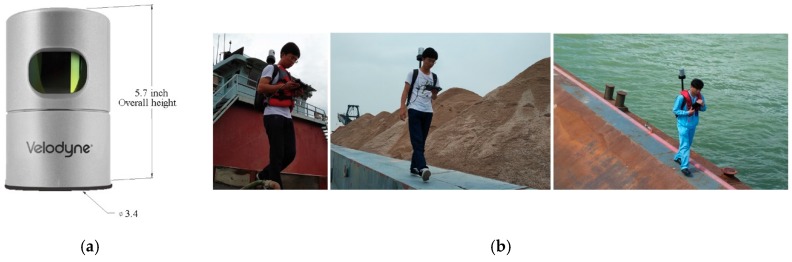
Velodyne LiDAR and laser scanning operation: (**a**) Velodyne HDL-32E LiDAR sensor; (**b**) laser scanning operation using the Velodyne HDL-32E LiDAR sensor.

**Figure 5:**
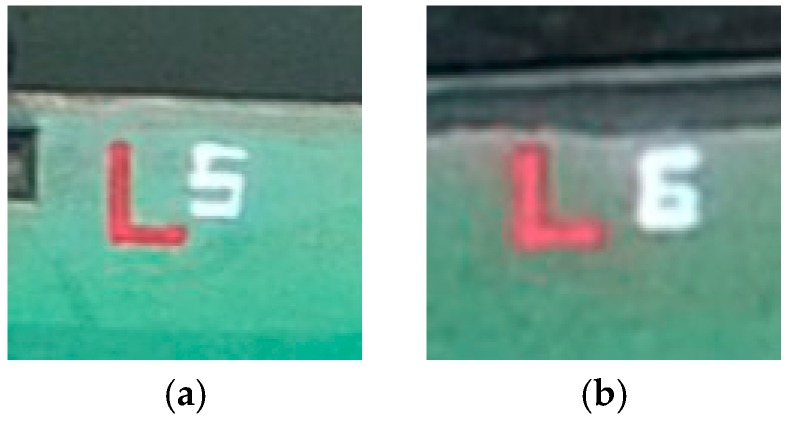
Two ground control points (GCPs) on a barge: (**a**) a GCP numbered 5; (**b**) a GCP numbered 6.

(2) For the laser scanning-based method, a Velodyne LiDAR with HDL-32E LiDAR sensor, as shown in Figure 4a, is used to collect the 3D point clouds of the surface of the stockpiles because of the sensor’s small size of only 5.7 inches in height and 3.4 inches in diameter, light weight of less than 2 kg, and rugged build; it also features up to 32 lasers across a 40° vertical field of view [30]. The detailed technical parameters of the Velodyne HDL-32E LiDAR sensor (Velodyne, San Jose, CA, USA) are given in Table 1. The surveyor carries the sensor on his back and walks along the side of the barge cabin to scan the stockpile-covered and stockpile-free surfaces with 0.7 million laser beams per second. The barges should basically be stationary and motionless during laser scanning; otherwise, the measured 3D point clouds become invalid. In other words, laser scanning cannot be used to reconstruct the surface of stockpiles when barges are moving or shaking.

(3) For UAV-based photogrammetry, the field measurement, i.e., GCP measurements, is conducted using a real-time kinematic GPS for approximately 20 min. Five GCPs for each of the experimental barges are measured for absolute orientation, and seven GCPs are measured as checkpoints to validate the accuracy of stockpile-covered and stockpile-free surface models. Finally, two GCPs are selected for exhibition in Figure 5. The validity of the GCPs measured is ensured by conducting the field measurement under a windless environment and on a static barge. The GCPs are used only for evaluation in this study; they are not actually needed for GCP-free UAV photogrammetry.

### 2.3. UAV-Based Video Acquisition

**Figure 6:**
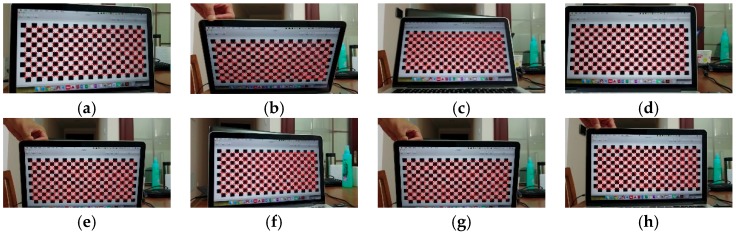
Eight views of the 2D chessboard exhibited as examples. (**a**–**h**) are the eight views of the 2D chessboard. Red circles with a center denote the referenced corners.

Generally, UAV-based stereo remotely sensed images are acquired in autonomous flights with waypoints predefined using the mission planning software package [19,20]. However, this method cannot satisfy the requirement of overlapping images using fixed waypoints when barges are moving or shaking. In this case, such images are extracted from UAV-based videos instead of images to ensure sufficient overlapping. Given the low-cost and flexible operation of quadcopters, this study uses a small quadcopter (DJI Mavic Pro, DJI, Shenzhen, China). It can capture high-resolution true-color videos through a 1/2.3 inch complementary metal-oxide-semiconductor (CMOS) sensor consumer-grade camera, and has a field of view of 78.8° and a focal length of 28 mm (35 mm format equivalent) [31]. The DJI Mavic Pro maintains the nadir orientation of the consumer-grade camera during video acquisition. UAV videos are obtained under good weather conditions, e.g., clear and sunny and winds <10 m/s. The flight altitude is set as 35 m above the barge level, and the ground sample distances are 2.3 cm/pix. The flight speed is set to 5 m/s, and the flying time is less than 1 min for each barge. The overlapping UAV-based remotely sensed images are extracted from the UAV-based video in a frame interval. The interior orientation parameters of the sensor carried on the DJI Mavic Pro are calculated from several views of a calibration pattern, i.e., the 2D chessboard pattern exhibited in Figure 6, in a relative reference system to minimize the systematic errors from the sensor. Systematic errors, i.e., distortions, can be modeled using a polynomial of four coefficients, e.g., two radial and two tangential distortion coefficients. The mean reprojected error of the adjustment is 0.46 pixels during the solution of the sensor parameters, which are listed in Table 2. The parameters are optimized through self-calibrating bundle adjustment.

## 3. Method

**Figure 7:**
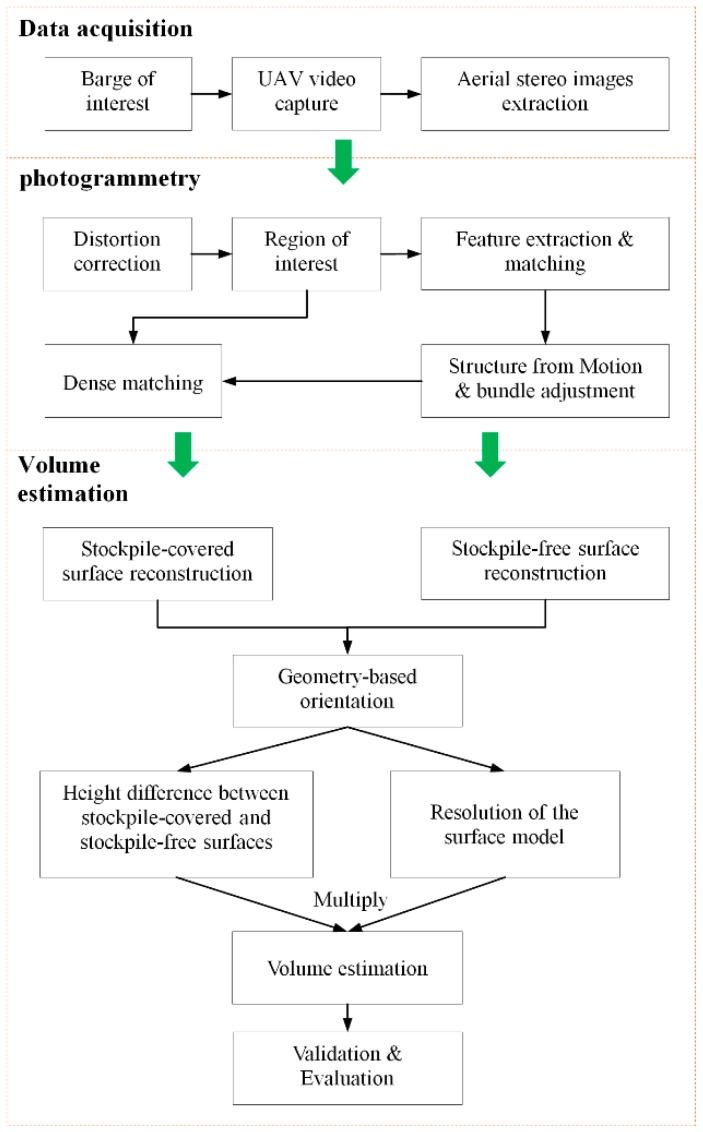
Workflow of the volume estimation of stockpiles carried on barges using GCP-free unmanned aerial vehicle (UAV) photogrammetry.

This study aims to use a workflow for the volume estimation of stockpiles carried on barges using UAV photogrammetry without the assistance of GCPs. The proposed approach, as demonstrated in Figure 7, includes four stages: (1) Self-adaptive stereo images are extracted to obtain overlapping images from UAV-based video. (2) Photogrammetric methods are used to generate 3D point clouds and reconstruct the stockpile-covered and stockpile-free surfaces through a series of steps, i.e., ROI-based image matching, SfM, bundle adjustment, and SGM. (3) A custom-built framework is established on the basis of the physical and geometric structure of the vessel and used to transform the stockpile-covered and stockpile-free surface models into a unified local spatial reference framework for volume estimation through the rotation transformations, i.e., tilt and plane rotations. (4) The volume of the stockpile is estimated by multiplying the height difference between the stockpile-covered and stockpile-free surface models by the size of the grid that is defined using the resolution of these models.

### 3.1. Aerial Stereo Images Extraction

**Figure 8:**
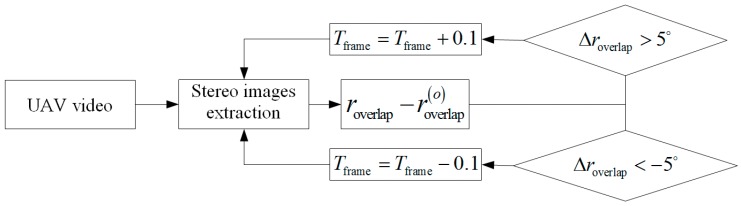
Validation of overlap for stereo image extraction.

In this study, UAV-based video is captured to ensure sufficient overlap because it can obtain a sequence of frames. The overlap of aerial stereo images is set to 80% to ensure sufficient overlaps in case of large fluctuations of the stockpile surface. On the basis of the variables, i.e., above the barge level ABL, flight speed $s_{\mathrm{UAV}}$, and focal length f of the sensor, the frame sampling interval $T_{\mathrm{frame}}$ is initially set using the following formula:

(2) $$T_{\mathrm{frame}}=\frac{w_{\mathrm{CMOS}}ABL\left(1-r_{\mathrm{overlap}}^{\left(o\right)}\right)}{s_{\mathrm{UAV}}f},$$

where $w_{\mathrm{CMOS}}$ denotes the width of the CMOS; $r_{\mathrm{overlap}}^{\left(o\right)}$ is the value of the given overlapping rate. Ideally, the flight speed is assumed to be a fixed value. However, it is difficult to maintain a constant speed under manual operation due to the influences of the operator’s operating level or external environmental factors, such as wind and barge moving speed. Therefore, as shown in Figure 8, overlap is validated to extract the stereo images with an overlap of approximately 80% and an effective solution in cases of the UAV slowing down or speeding up during data acquisition. The steps are as follows:

Step 1: Extract the stereo images from the UAV-based video in terms of the frame interval $T_{\mathrm{frame}}$.

Step 2: Match the stereo images using the SIFT algorithm on top of the image pyramid, then calculate the overlap $r_{\mathrm{overlap}}$ of the extracted stereo images and compute the overlapping difference $\Delta r_{\mathrm{overlap}}$ between the overlap $r_{\mathrm{overlap}}$ and the given overlap $r_{\mathrm{overlap}}^{\left(o\right)}$.

Step 3: Update $T_{\mathrm{frame}}=T_{\mathrm{frame}}+0.1$ when $\Delta r_{\mathrm{overlap}}$ is more than 5°; otherwise, update $T_{\mathrm{frame}}=T_{\mathrm{frame}}-0.1$ when $\Delta r_{\mathrm{overlap}}$ is less than −5°.

Step 4: Repeat Steps 1, 2, and 3 until the stereo images with an overlap of approximately 80% are extracted.

### 3.2. ROI-based Photogrammetry

Typically, UAV-based image acquisition from low-cost UAVs (e.g., DJI quadcopters) has large perspective distortions and poor sensor geometry [20,32], i.e., systematic errors, which need to be eliminated in terms of sensor parameters (Table 2).

**Figure 9:**
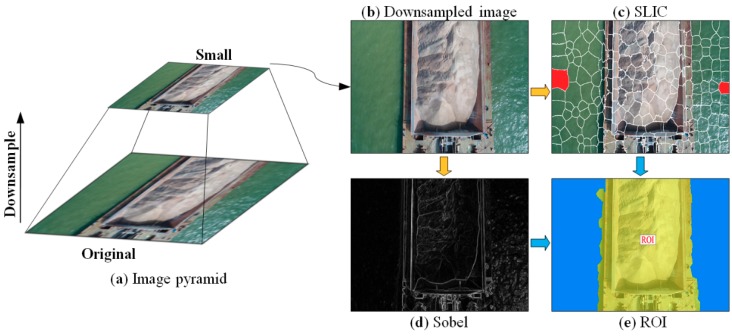
Region of interest (ROI) extraction on the top of the image pyramid by jointly using simple linear iterative clustering (SLIC) and Sobel algorithms: (**a**) UAV image pyramid; (**b**) the down-sampled image; (**c**) the result of SLIC segmentation in which two red superpixels are selected as seeds; (**d**) the gradient information detected using the Sobel algorithm; (**e**) the ROI, where blue and yellow denote the regions of water and barge, respectively.

The ROI of the barges and the stockpiles is defined to exclude the area of water in all UAV images and suit the volume measurement of the stockpiles carried on barges, thereby improving the accuracy of image matching and accelerating photogrammetry. In accordance with the clear gap of image color, intensity, and texture between barge and water, image segmentation is used to classify water and non-water regions. Moreover, effective and efficient segmentation is achieved by segmenting the UAV image on top of the pyramid (Figure 9a,b) into superpixels (Figure 9c) by a simple linear iterative clustering (SLIC) algorithm, which does not require much computational cost [33]. Mathematically, a down-sampled UAV image ${\mathbb{R}}_{s}^{w\times h}$ can be split into M superpixels, i.e., $I_{s}$ becomes a set of superpixels $\left\{R_{m}|m\in M\right\}$, where $R_{m}$ denotes the region of a superpixel m.

As shown in Figure 9b, the regions of water ${\mathbb{R}}^{\mathrm{water}}$ in a UAV image have relatively homogenous characteristics compared with the barge and thus can be used to exclude the superpixels within the water and create a mask of the barge. The region of barge ${\mathbb{R}}^{\mathrm{sgv}}$ can be calculated by excluding ${\mathbb{R}}^{\mathrm{water}}$ as:

(3) $${\mathbb{R}}^{\mathrm{sgv}}\leftarrow {\mathbb{R}}_{s}^{w\times h}-{\mathbb{R}}^{\mathrm{water}}.$$

The operation of two adjacent regions $R_{k}$ and $R_{l}$, which are merged into a new region is defined as:

(4) $$f\left(R_{k}\cup R_{l}\right)=f\left(R_{k}\right)⊕f\left(R_{l}\right),$$

where ⊕ denotes some operation on the values of $R_{k}$ and $R_{l}$. Let $f\left(R_{k}\right)=\left(N_{k},\mu_{k},c_{k}\right)$, in which

(5) $$\begin{array}{l} N_{k}=\left|R_{k}\right|,\\ \mu_{k}=\frac{1}{N_{k}}\sum_{i\in R_{k}}g_{i},\\ c_{k}=\frac{1}{N_{k}}\sum_{i\in R_{k}}X_{i}, \end{array}$$

where $N_{k}$, $\mu_{k}$, and $c_{k}$ are the number, color means, and regional centers of superpixel k, respectively; g denotes color values in red, green, and blue channels, respectively. Then, a new region $R_{\mathrm{new}}$ is generated, and the corresponding $N_{\mathrm{new}}$, $\mu_{\mathrm{new}}$, and $c_{\mathrm{new}}$ are updated as follows:

(6) $$\begin{array}{l} N_{\mathrm{new}}=N_{k}+N_{l},\\ \mu_{\mathrm{new}}=\frac{N_{k}\mu_{k}+N_{l}\mu_{l}}{N_{\mathrm{new}}},\\ c_{\mathrm{new}}=\frac{N_{k}c_{k}+N_{l}c_{l}}{N_{\mathrm{new}}}. \end{array}$$

The merging criteria of $R_{k}$ and $R_{l}$ can be defined using a similarity distance $d_{k,l}$, which is formed by a weighted combination of color mean distance $d_{k,l}^{\left(\mu \right)}$, region center distance $d_{k,l}^{\left(c\right)}$, and connectivity $d_{k,l}^{\left(b\right)}$ as follows:

(7) $$d_{k,l}\left(R_{k},R_{l}\right)=\alpha d_{k,l}^{\left(\mu \right)}+\beta d_{k,l}^{\left(c\right)}+\gamma d_{k,l}^{\left(b\right)},$$

where α, β, and γ are the weights corresponding to $d_{k,l}^{\left(\mu \right)}$, $d_{k,l}^{\left(c\right)}$, and $d_{k,l}^{\left(b\right)}$, respectively, and are computed as:

(8) $$\begin{array}{l} d_{k,l}^{\left(\mu \right)}=\frac{N_{k}}{N_{\mathrm{new}}}{\left|\mu_{k}-\mu_{\mathrm{new}}\right|}^{2}+\frac{N_{l}}{N_{\mathrm{new}}}{\left|\mu_{l}-\mu_{\mathrm{new}}\right|}^{2},\\ d_{k,l}^{\left(c\right)}=\frac{N_{k}}{N_{\mathrm{new}}}{\left|c_{k}-c_{\mathrm{new}}\right|}^{2}+\frac{N_{l}}{N_{\mathrm{new}}}{\left|c_{l}-c_{\mathrm{new}}\right|}^{2},\\ d_{k,l}^{\left(b\right)}=1-\frac{L_{\mathrm{weak}}}{L}, \end{array}$$

where L denotes the common boundary between $R_{k}$ and $R_{l}$; $L_{\mathrm{weak}}$ is the length of the weak part of the boundary and can be determined by gradient intensity on the boundary (Figure 9d), which is computed by using Sobel operator. Generally, UAV images contain a part of water regions on both sides of the barge to ensure the coverage of full sides. Thus, only one strip of overlapping UAV images can cover a barge. In this case, two red superpixels on both sides of the UAV image in Figure 9c are selected as seeds to trigger superpixel merging. Then, the ROI is shaped by reversing the water regions using Equation (3).

Feature extraction and matching are performed using a sublevel Harris operator (S-Harris) coupled with the SIFT algorithm, which is the most popular and commonly used method in the field of photogrammetry and computer vision [34,35]. To achieve evenly distributed matches, this study uses a coarse-to-fine matching strategy to find corresponding points between two stereo images under the constraint of the ROI instead of directly matching images using SIFT. In the coarse-matching stage, ROI-based SIFT feature extraction and matching on the top of the UAV image pyramid are performed to compute the initial relative orientation of two stereo images. In the fine-matching stage, gridding S-Harris operator [28] and SIFT descriptor (S-Harris-SIFT) are jointly used to find the corresponding points along the epipolar lines obtained from the initial relative orientation. Clearly, these stages are implemented to accelerate the efficiency and accuracy of image matching, and especially to obtain evenly distributed matches even in areas with weak texture.

Traditionally, aerial triangulation is assisted by the initial exterior orientation parameters obtained from an airborne GPS and inertial measurement unit. Compared with traditional photogrammetry, the exterior orientation parameters of each frame in the UAV video cannot be captured and cannot be available for aerial triangulation. Fortunately, SfM is suited to recover 3D geometry from the stereo images captured from the video because it can allow 3D reconstruction without the assistance of exterior orientation parameters. Self-calibrating bundle adjustment (i.e., using sparse bundle adjustment software [36]) is also conducted to optimize 3D points and interior (Table 2) and exterior orientation parameters. In addition, the SGM algorithm is utilized to reconstruct the fine details of the stockpile surface by dense matching within the ROI.

**Figure 10:**
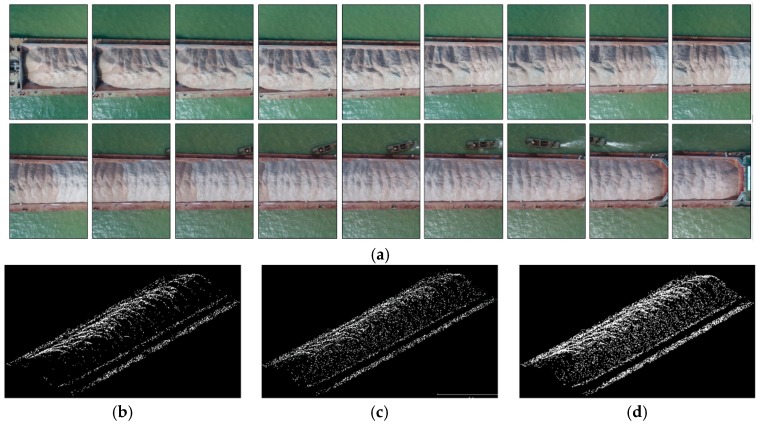

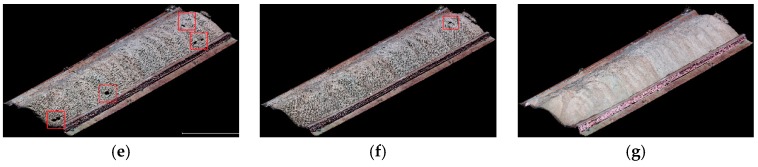
Results of sparse and dense matching in 3D space: (**a**) the overlapping images used; (**b**), (**c**), and (**d**) are the sparse point clouds obtained using SIFT, non-ROI-based matching, and ROI-based matching, respectively; (**e**), (**f**), and (**g**) are the dense point clouds corresponding to the results of (**b**), (**c**), and (**d**); the subregions marked by red are the low-quality 3D points.

A sequence of the stereo images extracted from the UAV video is selected to evaluate the 3D reconstruction of the stockpile using SIFT, non-ROI-based coarse-to-fine matching (i.e., non-ROI-based matching), and ROI-based coarse-to-fine matching (i.e., ROI-based matching). The visualization results are exhibited in Figure 10, and the number of matches is summarized in Table 3. Results show that denser matches with higher quality can be obtained using ROI-based matching compared with using SIFT and non-ROI-based matching. The root mean square error (RMSE) statistics summarized in Table 4 are calculated on the basis of the seven checkpoints and their corresponding 3D points measured using the digital surface model (DSM). On this basis, 3D reconstruction using ROI-based matching is performed better than by using SIFT and non-ROI-based matching in terms of the number and quality of matches and RMSE values. As shown in Table 4, the horizontal (i.e., X and Y) and vertical (i.e., Z) RMSEs obtained by ROI-based matching are approximately equal to 4 and 5 cm, respectively. Thus, these RMSE values seem relatively satisfactory and sufficient to estimate the volume of the stockpiles carried on the barges. In addition, during the computational efficiency analysis, ROI-based matching also shows a significant improvement in the computational cost of approximately one-fifth the time consumed by SIFT. Furthermore, even though ROI-based matching increases the computational cost of ROI detection, it requires slightly more time compared with non-ROI-based matching due to the small searching scope of the matches.

### 3.3. Custom-Built Framework

Generally, absolute orientation is an essential step to transform the photogrammetric point clouds in an arbitrary coordinate system into a ground coordinate system using several GCPs. However, the generic method of absolute orientation is only suitable for static ground objects and not for stockpiles in a dynamic environment. Importantly, it cannot be ignored here as the aim is to align stockpile-covered and stockpile-free surface models for volume estimation. In comparison with topographic surveys in which all photogrammetric point clouds need to be transformed into a unified geographic coordinate system, only the stockpile-covered and stockpile-free surface models need to be transformed into a local spatial framework for volume estimation in the present study. Furthermore, a custom-built framework is established to transform the stockpile-covered and stockpile-free surface models into a local spatial coordinate system based on the geometry of the vessel instead of the requirement of GCP measurement or the georeferencing coordinate system. The coordinate transformation can be represented as follows:

(9) $$\left[\begin{array}{c} x\\ y\\ z \end{array}\right]=\lambda R\left[\begin{array}{c} x^{\prime }\\ y^{\prime }\\ z^{\prime } \end{array}\right]+T,$$

where ${\left[\begin{array}{ccc} x & y & z \end{array}\right]}^{T}$ and ${\left[\begin{array}{ccc} x^{\prime } & y^{\prime } & z^{\prime } \end{array}\right]}^{T}$ are the coordinates in the custom-built framework and the auxiliary coordinate system, respectively; R and T are the rotation and translation matrices, respectively; λ denotes a scale factor.

**Figure 11:**
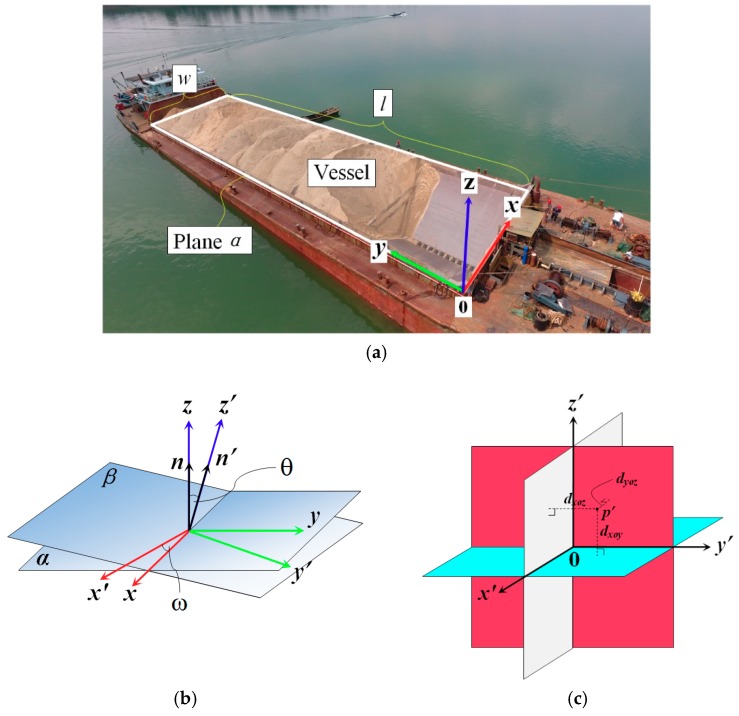
Custom-built framework: (**a**) a custom-built framework that is established using a horizontal plane α with known length l and width w; (**b**) the spatial geometric rotation between the custom-built coordinate system *O-XYZ* and an auxiliary coordinate system *O-X′Y′Z′;* (**c**) the transformation of a photogrammetric point p′ from the arbitrary coordinate system to the auxiliary coordinate system.

As exhibited in Figure 11a, assuming a horizontal plane α with a known length l and width w of a vessel can be extracted to establish a local planar coordinate system *XOY*, the four corners located on the rectangle can be used to define four coordinates in the custom-built framework. In other words, (0,0,0), (w,0,0), (w,l,0), and (0,l,0), and the corresponding coordinates $\left(x_{i}^{\left(a\right)},y_{i}^{\left(a\right)},z_{i}^{\left(a\right)}|i\in \left\{1,2,3,4\right\}\right)$ in the arbitrary coordinate system can be measured using the stockpile-covered and stockpile-free surface models. The normal vector n=(0,0,1) of this plane is considered the axis *Z*, i.e., a custom-built coordinate system is established into *O-XYZ*. The photogrammetric point clouds with the arbitrary coordinate system are transformed into an auxiliary coordinate system *O-X′Y′Z′*, which is established on the basis of the geometry and normal vector $n^{\prime }$ of plane β, which are defined using the coordinates $\left[x_{i}^{\left(a\right)},y_{i}^{\left(a\right)},z_{i}^{\left(a\right)}\right]\left(i\in \left\{1,2,3,4\right\}\right)$ in the arbitrary coordinate system. The coefficients {A,B,C,D} of plane β can be solved by listing four systems of equations as follows:

(10) $$Ax_{i}^{\left(a\right)}+By_{i}^{\left(a\right)}+Cz_{i}^{\left(a\right)}+D=0.$$

As shown in Figure 11b, the normal vector $n^{\prime }=\left(A,B,C\right)$ of this plane is considered the axis *Z′*. Axis *X′* can be defined on the basis of the cross product of the normal vectors $n^{\prime }$ and n, and then axis *Y′* is defined on the basis of the cross product of axes *Z′* and *X′*. Subsequently, each 3D photogrammetric point with the arbitrary coordinate system can be transformed into the auxiliary coordinate system *O-X′Y′Z′* according to the distance from the point to each plane in the auxiliary coordinate system *O-X′Y′Z′*, e.g., $d_{yoz}$, $d_{xoz}$, and $d_{xoy}$. As shown in Figure 11c, the coordinate of a point $p^{\prime }$ in the auxiliary coordinate system *O-X′Y′Z′* is $\left[d_{yoz},d_{xoz},d_{xoy}\right]$, which can be computed as:

(11) $$\{\begin{array}{c} d_{yoz}=\frac{\left|A_{yoz}x^{\prime }+B_{yoz}y^{\prime }+C_{yoz}z^{\prime }+D_{yoz}\right|}{\sqrt{A_{yoz}{}^{2}+B_{yoz}{}^{2}+C_{yoz}{}^{2}}}\\ d_{xoz}=\frac{\left|A_{xoz}x^{\prime }+B_{xoz}y^{\prime }+C_{xoz}z^{\prime }+D_{xoz}\right|}{\sqrt{A_{xoz}{}^{2}+B_{xoz}{}^{2}+C_{xoz}{}^{2}}}\\ d_{xoy}=\frac{\left|A_{xoy}x^{\prime }+B_{xoy}y^{\prime }+C_{xoy}z^{\prime }+D_{xoy}\right|}{\sqrt{A_{xoy}{}^{2}+B_{xoy}{}^{2}+C_{xoy}{}^{2}}} \end{array},$$

where $\left[x^{\left(a\right)},y^{\left(a\right)},z^{\left(a\right)}\right]$ is the coordinate of the point $p^{\prime }$ in the arbitrary coordinate system; $\left\{A_{yoz},B_{yoz},C_{yoz},D_{yoz}\right\}$, $\left\{A_{xoz},B_{xoz},C_{xoz},D_{xoz}\right\}$, and $\left\{A_{xoy},B_{xoy},C_{xoy},D_{xoy}\right\}$ are the coefficients of planes *YOZ*, *XOZ*, and *XOY*, respectively. Then, the scale factor of Equation (9) can be computed with the centralization of coordinates as follows:

(12) $$\begin{array}{l} \lambda =\frac{1}{n}\sum_{i=1}^{n}\frac{\sqrt{{\bar{x}}_{i}{}^{2}+{\bar{y}}_{i}{}^{2}+{\bar{z}}_{i}{}^{2}}}{\sqrt{{\bar{x}}_{i}^{\prime }{}^{2}+{\bar{y}}_{i}^{\prime }{}^{2}+{\bar{z}}_{i}^{\prime }{}^{2}}},\\ {\bar{x}}_{i}=x_{i}-\frac{1}{n}\sum_{i=1}^{n}x_{i},{\bar{y}}_{i}=y_{i}-\frac{1}{n}\sum_{i=1}^{n}y_{i},{\bar{z}}_{i}=z_{i}-\frac{1}{n}\sum_{i=1}^{n}z_{i},\\ {\bar{x}}_{i}^{\prime }=x_{i}^{\prime }-\frac{1}{n}\sum_{i=1}^{n}x_{i}^{\prime },{\bar{y}}_{i}^{\prime }=y_{i}^{\prime }-\frac{1}{n}\sum_{i=1}^{n}y_{i}^{\prime },{\bar{z}}_{i}^{\prime }=z_{i}^{\prime }-\frac{1}{n}\sum_{i=1}^{n}z_{i}^{\prime }, \end{array}$$

where n is the number of known coordinates, which is set to 4 in terms of the number of corners on the barge in this study; $\left({\bar{x}}_{i},{\bar{y}}_{i},{\bar{z}}_{i}\right)$ and $\left({\bar{x}}_{i}^{\prime },{\bar{y}}_{i}^{\prime },{\bar{z}}_{i}^{\prime }\right)$ are the centralized coordinates in the coordinate systems *O-XYZ* and *O-X′Y′Z′*, respectively. Then, the transformation of the two coordinates using Equation (9) can be expressed using the following equation, and the rotation matrix R can be computed as:

(13) $$\left[\begin{array}{c} {\bar{x}}_{i}\\ {\bar{y}}_{i}\\ {\bar{z}}_{i} \end{array}\right]=\lambda R\left[\begin{array}{c} {\bar{x}}_{i}^{\prime }\\ {\bar{y}}_{i}^{\prime }\\ {\bar{z}}_{i}^{\prime } \end{array}\right].$$

As shown in Figure 11b, the rotation matrix that consists of a tilt and plane rotation can be computed as:

(14) $$R=R\left(\theta \right)\cdot R\left(\omega \right)=\left[\begin{array}{ccc} 1 & 0 & 0\\ 0 & \cos\theta & -\sin\theta \\ 0 & \sin\theta & \cos\theta \end{array}\right]\left[\begin{array}{ccc} \cos\omega & -\sin\omega & 0\\ \sin\omega & \cos\omega & 0\\ 0 & 0 & 1 \end{array}\right]=\left[\begin{array}{ccc} \cos\omega & -\sin\omega & 0\\ \cos\theta \sin\omega & \cos\theta \cos\omega & -\sin\theta \\ \sin\theta \sin\omega & \sin\theta \cos\omega & \cos\theta \end{array}\right],$$

where the tilt rotation angle θ can be computed as:

(15) $$\theta =\cos^{-1}\left(\frac{nn^{\prime }}{\left|n\right|\left|n^{\prime }\right|}\right).$$

Next, the plane rotation angle ω is computed using Equation (14) and the known rotation matrix R. Then, the translation matrix T is computed using Equation (9). Therefore, the geometry structure of a barge can be utilized to establish a custom-built framework for defining a local reference between stockpile-covered and stockpile-free surface models instead of requiring a prerequisite of GCP measurement. The superiority of the custom-built framework is that it can make GCP-free UAV photogrammetry work well for 3D reconstruction with the physical object size regardless if a barge is moving or not.

### 3.4. Surface Modeling and Volume Estimation

A photogrammetric point of a stockpile in the 3D custom-built space can be represented using an O×3 matrix $P\left(p_{x},p_{y},p_{z}\right)$. Accordingly, all the 3D photogrammetric points can be converted into an *M*-by-*N* matrix filled by the height values, where *M* and *N* are the number of 3D points in the directions of length and width, respectively. Generally, the objective of surface modeling is to create a mathematical function or use an interpolation algorithm from the point clouds to approximate the true stockpile surface. The execution time of surface modeling often needs to meet the real-time modeling requirement. Nonetheless, fitting the stockpile surface from such a dense point cloud is time consuming. UAV photogrammetry can generate sufficient dense point clouds (e.g., 3.5 cm/pix in this study). Thus, a good trade-off between modeling time and accuracy is expected using the *M*-by-*N* matrix to represent the 3D surface of the stockpile instead of some complex interpolation methods. In addition, noise filtering of the 3D surface is achieved using the difference of the Gaussian and moving surface functions [20]. Subsequently, the volume $V_{\mathrm{stockpile}}$ of the stockpiles carried on the barges is calculated by the height difference between the stockpile-covered and stockpile-free surface models multiplied by the size of the grid that is defined using the resolution of these models. Then, volume estimation can be computed as:

(16) $$V_{\mathrm{stockpile}}=\iint \left(H_{\mathrm{covered}}-H_{\mathrm{free}}\right)dldw,$$

where $H_{\mathrm{covered}}$ and $H_{\mathrm{free}}$ are the height values of stockpile-covered and stockpile-free surface models, respectively; l and w are the length and width of the vessel.

## 4. Results and Discussion

In the experiments, stockpiles carried on eight barges (half moving and half non-moving barges in the UAV video acquisition) are used to evaluate the proposed method, which is compared with the traditional manual measurement. In addition, the other point clouds collected from a different source, i.e., laser scanning, are also used to compare and examine how close the numbers obtained from GCP-free UAV photogrammetry are. Traditionally, the volume of a regularly shaped stockpile that is estimated using the trapezoidal method is considered to be accurate and acceptable.

**Figure 12:**
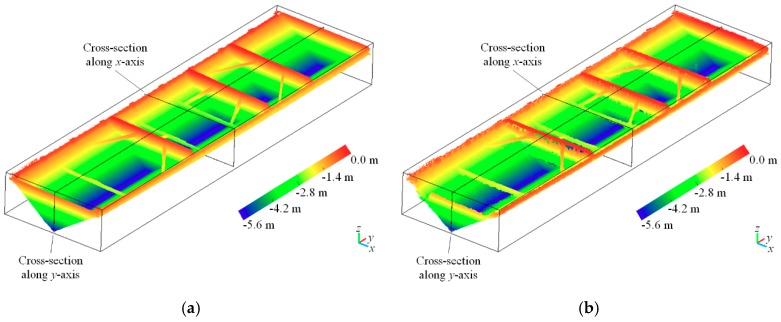

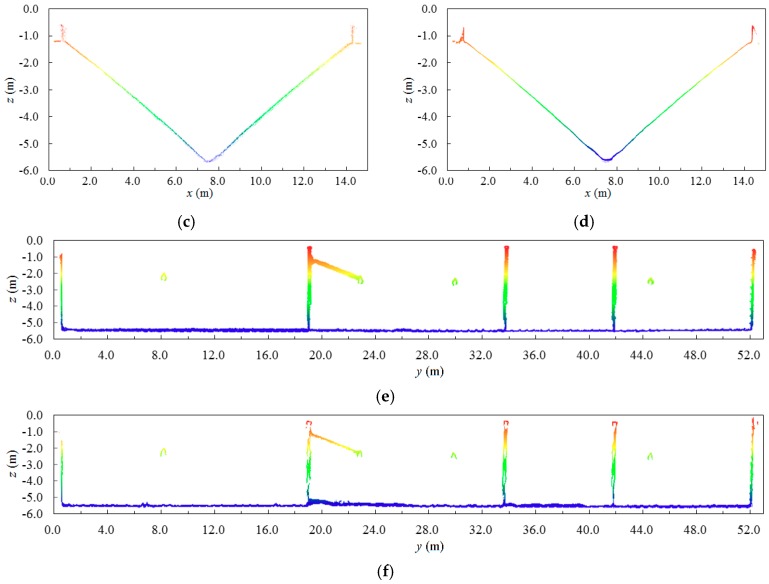
Comparisons of point clouds generated from laser scanning and UAV photogrammetry for stockpile-free modeling: (**a**) and (**b**) are the point clouds generated from laser scanning and UAV photogrammetry, respectively; (**c**) and (**d**) are the respective cross sections of (**a**) and (**b**) along the x-axis; (**e**) and (**f**) are the respective cross sections of (**a**) and (**b**) along the y-axis.

**Figure 13:**
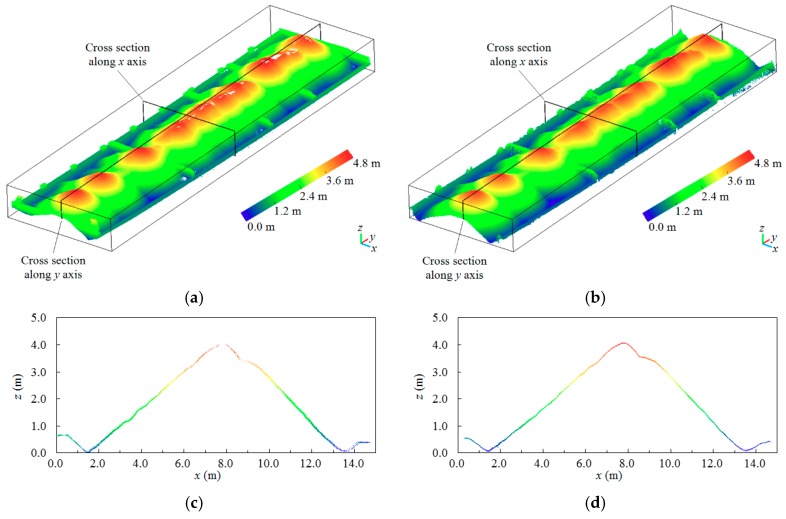

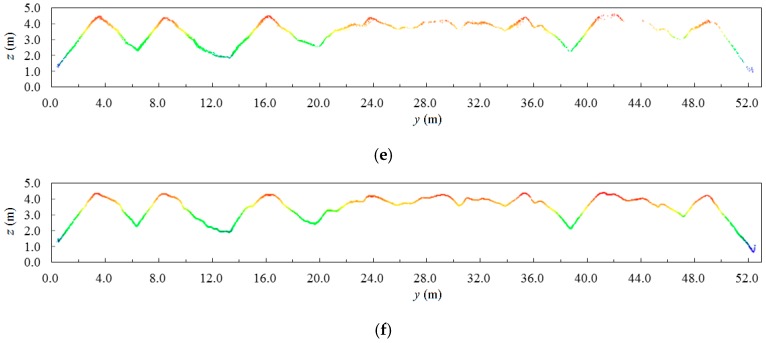
Comparisons of point clouds generated from laser scanning and UAV photogrammetry for stockpile-covered modeling: (**a**) and (**b**) are the point clouds generated from laser scanning and UAV photogrammetry, respectively; (**c**) and (**d**) are the respective cross sections of (**a**) and (**b**) along the x-axis; (**e**) and (**f**) are the respective cross sections of (**a**) and (**b**) along the y-axis.

One of the eight barges, including the point clouds of stockpile-covered and stockpile-free surfaces, is shown in Figure 12 and Figure 13. The visualization results show that the UAV photogrammetric point clouds are close to the LiDAR point clouds obtained by laser scanning, and these point clouds obtained from the different sources can represent the finely detailed stockpile-covered and stockpile-free surfaces. As shown in Figure 12, laser scanning can acquire all the interior side structures of the stockpile-free surface (i.e., vessel), although acquiring these structures may not necessarily be required for the proposed volume estimation, which depends on the height values. The cross section along the y-axis within the point clouds of the stockpile-free surface obtained by laser scanning is closer to the true stockpile-free surface structure than that obtained by UAV photogrammetry; nevertheless, the difference is not evident. In the case where the stockpile is stacked beyond the angle of laser scanning, which may not be able to collect complete 3D points at the top of the stockpile-covered surface, the missing areas of the point clouds are filled using B-spline interpolation, as shown in Figure 13a,e and [4]. In addition, the cross sections along the x and y axes in Figure 13 also exhibit similar stockpile-covered surfaces obtained by laser scanning and UAV photogrammetry. In the experiments, the UAV-derived and LiDAR point clouds, including four out of the eight barges (i.e., nonmoving barges), are absolutely oriented using the five measured GCPs. The RMSE is calculated on the basis of the seven GCPs and their corresponding 3D points measured using the stockpile-covered and stockpile-free surface models. The error statistics are summarized in Table 5. The X and Y RMSE values, which are slightly different between UAV photogrammetry and laser scanning, are approximately 5 cm. The vertical RMSE values of the two methods are less than 8 cm, although laser scanning demonstrates better accuracy in the vertical RMSE compared with UAV photogrammetry. The RMSE values of the UAV-based method remain to be relatively satisfactory and sufficient to estimate the volume of stockpiles carried on barges.

Table 6 shows the volume measured by traditional manual measurement, laser scanning, and UAV photogrammetry. Correspondingly, volume estimation is also performed using UAV photogrammetry with GCPs (B1−B4) and without (B5−B8). The deviation between the volume estimated by laser scanning and UAV photogrammetry and the volume calculated by traditional manual measurement is relatively small and approximately equal to ±2%, which can be considered acceptable because the volume is often calculated with a precision of ±3% accuracy of the whole amount [11,16]. Inevitably, errors occur in volume calculation using the traditional method, and providing a completely correct volume for comparison is difficult. Benefiting from the dense point clouds obtained from laser scanning and UAV photogrammetry, the stockpile-covered and stockpile-free surfaces can be accurately detailed, thereby providing results similar to that of the traditional method. Thus, results suggest that laser scanning (in a non-dynamic environment) and UAV photogrammetry can be used as an effective alternative to traditional manual measurement for the volume estimation of stockpiles carried on barges.

Although all three methods may accurately calculate the volume of stockpiles, the proposed approach using GCP-free UAV photogrammetry is considered the most suitable for a dynamic environment because of four reasons. First, the traditional measurement calculates volume by manually shaping the stockpiles into regular shapes (e.g., trapezoid). This method is costly and time consuming. Furthermore, a perfectly regular shape of stockpiles is difficult to obtain, and the local details of stockpiles cannot be obtained. Therefore, the quality of stockpile shaping is difficult to control. By contrast, laser scanning and UAV photogrammetry can provide dense point clouds to reconstruct the stockpile-covered and stockpile-free surfaces accurately. Second, the surveyor needs to walk around the vessel as smoothly as possible in the course of laser scanning and to avoid the laser sensor swaying excessively; otherwise, the point clouds cannot be fitted, especially in vessel corners and stairs. In addition, when a stockpile is stacked beyond the angle of laser scanning, the surveyor needs to climb the stockpile to scan the surface. Thus, the instability of walking on the top of the stockpile may cause laser sensor vibration and generate invalid data. Some invalid data are generated because of the shaking of the laser sensor during the experiments. Moreover, the point clouds of the stockpile cannot be obtained under the condition of a moving and shaking barge. In this case, UAV photogrammetry can still perform data acquisition regardless of if the barge is moving or not. Third, the traditional method and laser scanning requires boarding for survey operations and may result in health risks associated with exposing surveyors to danger during on-site operations; meanwhile, UAV photogrammetry can be conducted from a long distance without boarding the barge and without touching the stockpile to collect data. In this study, GCP-free UAV photogrammetry using a custom-built framework does not need GCP field measurement; only the physical geometry structure of the vessel is needed to establish a local reference between the stockpile-covered and stockpile-free surface models. Fourth, GCP-free photogrammetry, which requires an average of only 20 min per barge for data acquisition and processing, is more efficient than the traditional method and laser scanning, which require 120 and 40 min, respectively. Overall, the results obtained by GCP-free UAV photogrammetry are approximately equal to those obtained by laser scanning, and the proposed approach has strong applicability to barges in a dynamic environment. The overall results suggest that GCP-free UAV photogrammetry can be used as an effective alternative to manual measurements for a rapid volume estimation of stockpiles carried on barges.

## 5. Conclusions

The present study aimed to estimate the volume of stockpiles carried on eight barges using low-cost and high-density 3D UAV-based photogrammetric point clouds. These point clouds were oriented by the geometric structure of the vessel instead of prerequisite GCPs. A UAV video with a ground sample distance of approximately 2.3 cm/pix was captured to extract stereo images for ensuring sufficient overlaps with large fluctuations of the stockpile surface. High-density 3D point clouds were generated to reconstruct the stockpile-covered and stockpile-free surfaces (e.g., 3.5 cm/pix in this study). The horizontal and vertical RMSE values of the stockpile-covered and stockpile-free surface models were approximately equal to 5 cm and less than 8 cm, respectively. A local spatial coordinate system based on the geometric structure of the vessel was also established to provide a reference between the stockpile-covered and stockpile-free surface models. A relatively small deviation of approximately ±2% between the volume estimated by UAV photogrammetry and the volume calculated by traditional manual measurement was obtained. The deviation can be considered acceptable in terms of the legislation statement, and it can satisfy the requirement of volume estimation of the stockpiles carried on the eight barges. Therefore, the overall results suggest that our method can be used as an effective alternative to the traditional method for estimating the volume of stockpiles carried on barges.

The custom-built framework established in this study assumes a horizontal plane on the vessel of the barge, which is only suitable for barges with a rectangular vessel. In some cases, the geometric structure of the vessel may be deformed rather than rigidly planar or rectangular, and then systematic errors may occur to reduce the accuracy of volume estimation. In future studies, we will attempt to construct a 3D custom-built framework to minimize the systematic errors caused by the deformation of the geometric structure of a barge.

## Figures and Tables

**Table 1:** Technical parameters of the Velodyne HDL-32E LiDAR sensor.

Parameters	Value
Maximum measuring distance	100 m
Channels	32
Accuracy	±2 cm
Field of view angle (vertical)	+10.67° to −30.67°
Angular resolution (vertical)	1.33°
Field of view angle (horizontal)	360°
Angular resolution (horizontal/azimuth)	0.1° to 0.4°
Rotation rate	5 to 20 Hz
Output	0.7 million points per second

**Table 2:** Parameters of the sensor carried on the DJI Mavic Pro. $f_{x}$ and $f_{y}$ are the focal lengths expressed in pixel units; (cx,cy) is a principal point that is usually at the image center; $k_{1}$ and $k_{2}$ are the radial distortion coefficients; $p_{1}$ and $p_{2}$ are the tangential distortion coefficients.

Parameters	Value (pixel)
Frame size	1920 × 1080
$f_{x}$	1537.42
$f_{y}$	1536.61
cx	917.71
cy	542.67
$k_{1}$	0.17256914
$k_{2}$	−0.82566273
$p_{1}$	0.00007309
$p_{2}$	−0.00528847

**Table 3:** Number of matches obtained using scale-invariant feature transform (SIFT), S-Harris-SIFT, and ROI-based S-Harris-SIFT, respectively.

Method	Matches	
	Sparse Matching	Dense Matching
SIFT	6330	237,411
non-ROI-based matching	8561	250,012
ROI-based matching	10,876	298,338

**Table 4:** Error statistics of checkpoints measured using DSMs. These errors are derived from differencing with checkpoints that serve as a reference. DSMs: digital surface models; RMSE: root mean square error.

Method	RMSE X (cm)	RMSE Y (cm)	RMSE Z (cm)	Total RMSE (cm)
SIFT	9.10	8.05	13.78	10.71
non-ROI-based matching	4.96	5.34	7.02	5.66
ROI-based matching	4.08	4.14	5.09	4.29

**Table 5:** Error statistics of GCPs measured using the stockpile-covered and stockpile-free surface models in a non-dynamic environment. These errors are derived from differencing with GCPs that serve as a reference. RMSE: root mean square error. B1–B4 denote the number of barges.

No.	Data Source	RMSE X (cm)	RMSE Y (cm)	RMSE Z (cm)	Total RMSE (cm)
B1	LiDAR	5.67	5.22	4.29	5.09
	UAV	4.32	5.83	7.72	5.81
B2	LiDAR	4.96	5.04	4.19	4.55
	UAV	5.52	5.05	7.33	5.83
B3	LiDAR	5.21	5.13	4.43	4.86
	UAV	4.53	4.39	6.13	4.89
B4	LiDAR	4.96	5.35	4.23	4.85
	UAV	4.08	4.14	5.09	4.29

**Table 6:** Comparisons of volume calculation using traditional manual measurement (Manual), laser scanning (LiDAR), and UAV photogrammetry (UAV). B1–B8 denote the number of barges; DE and NDE denote the case of dynamic and non-dynamic environments, respectively; Res. L.: the residuals between LiDAR and Manual; Res. U.: the residuals between UAV and Manual; Dev. L.: the percentage of deviation calculated by dividing Res. L. by Manual; Dev. U.: the percentage of deviation calculated by dividing Res. U. by Manual; NULL: the invalid data in the dynamic environment.

No.	Time	Case	Manual (m^3^)	LiDAR (m^3^)	UAV (m^3^)	Res. L. (m^3^)	Res. U. (m^3^)	Dev. L. (%)	Dev. U. (%)
B1	08/06/2018	NDE	2356.17	2315.41	2308.34	−40.76	−47.83	−1.73	−2.03
B2	08/06/2018	NDE	2173.44	2208.87	2200.61	35.43	27.17	1.63	1.25
B3	10/06/2018	NDE	2832.83	2779.57	2783.54	−53.26	−49.29	−1.88	−1.74
B4	10/06/2018	NDE	2074.23	2047.68	2053.90	−26.55	−20.33	−1.28	−0.98
B5	11/06/2018	DE	2662.10	NULL	2718.27	NULL	51.17	NULL	1.92
B6	11/06/2018	DE	2783.73	NULL	2829.10	NULL	45.37	NULL	1.63
B7	12/06/2018	DE	2431.41	NULL	2386.19	NULL	−45.22	NULL	−1.86
B8	12/06/2018	DE	2568.35	NULL	2531.62	NULL	−36.73	NULL	−1.43
